# Long-Term Response to Galantamine in Relation to Short-Term Efficacy Data: Pooled Analysis in Patients with Mild to Moderate Alzheimer’s Disease

**DOI:** 10.2174/156720511795256044

**Published:** 2011-03

**Authors:** S Kavanagh, I Howe, H R Brashear, D Wang, B Van Baelen, M Todd, S Schwalen

**Affiliations:** 1 Johnson & Johnson Pharmaceutical Services, Beerse, Belgium; 2 Shire Pharmaceuticals Limited, UK; 3 Johnson & Johnson Pharmaceutical Research and Development, LLC, Titusville, NJ, USA (Current address for H. Robert Brashear and Daniel Wang: Janssen Alzheimer Immunotherapy, South San Francisco, USA); 4 SGS Life Science Services, Mechelen, Belgium; 5 Johnson & Johnson Pharmaceutical Research and Development, LLC, Titusville, NJ, USA; 6 Janssen-Cilag GmbH, Neuss, Germany (Current address: Grünenthal GmbH, Aachen, Germany)

**Keywords:** Alzheimer’s disease, cognition, dementia, galantamine.

## Abstract

**Background:**

This analysis aimed to identify an operational, clinically relevant definition of response achieved in short-term clinical trials to support the identification of patients with Alzheimer’s disease (AD) who would benefit most from long-term galantamine therapy.

**Methods:**

Data were analyzed from 6 randomized placebo-controlled trials of up to 6 months’ duration, which included patients with mild to moderate AD receiving maintenance doses of galantamine 16-24 mg/day, and from 12 open-label extensions (galantamine 24 mg/day maintenance therapy). Assessments included changes from baseline in the 11-item AD Assessment Scale-Cognitive subscale (ADAS-Cog 11).

**Results:**

Pooled analysis of the 5-6 month trial data showed that at the trial endpoint (2-5 months after reaching maintenance doses), the proportions of galantamine- (n=1,173) versus placebo-treated patients (n=801) with probable AD categorized according to “improved”, “stable” or “non-rapid decline” criteria, were 45.8% versus 27.2%, 59.5% versus 37.1%, and 87.6% versus 69.7%, respectively (observed cases analysis), whilst changes in ADAS-Cog 11 scores versus baseline were -4.9, -4.7 and -2.9 points, respectively, for “improved”, “stable” and “non-rapid decline” galantamine-treated patients (-1.5 points for galantamine recipients overall). “Improved” or “stable” galantamine-treated patients displayed mean improvement in ADAS-Cog 11 scores over baseline until 18 months after starting treatment, and attenuated deterioration thereafter; for galantamine-treated patients exhibiting “non-rapid decline”, mean ADAS-Cog 11 score returned to baseline after approximately 12 months.

**Conclusions:**

Patients who demonstrate improvement, stability, or limited cognitive decline 2-5 months after reaching maintenance doses of galantamine are more likely to experience continued benefit from long-term galantamine therapy.

## INTRODUCTION

Galantamine is licensed for the treatment of mild to moderate Alzheimer’s disease (AD) and is administered at maintenance doses of 16 or 24 mg daily [1]. The short-term cognitive, functional and behavioral benefits of galantamine in patients with AD were established in double-blind, placebo-controlled randomized clinical trials (RCTs) of 3-6 months’ duration [2-7]. Similar short-term beneficial effects were reported for patients with AD and cerebrovascular disease (AD+CVD) [8]. In long-term, open-label extensions (up to 36 months), the cognitive decline observed in galantamine-treated patients with AD appeared attenuated versus epidemiologically based predictions of untreated decline [4, 9-11]. Furthermore, it has been estimated that each additional year of galantamine treatment is associated with a 27% reduction in the relative risk of permanent admission to a residential or nursing home [12].

The present analysis aimed to identify an operational, clinically relevant definition of response achieved in short-term clinical trials that could aid clinicians in identifying patients with AD who would benefit most from long-term galantamine therapy.

## MATERIALS AND METHODS

### Data Sources

**Fig. (1):**
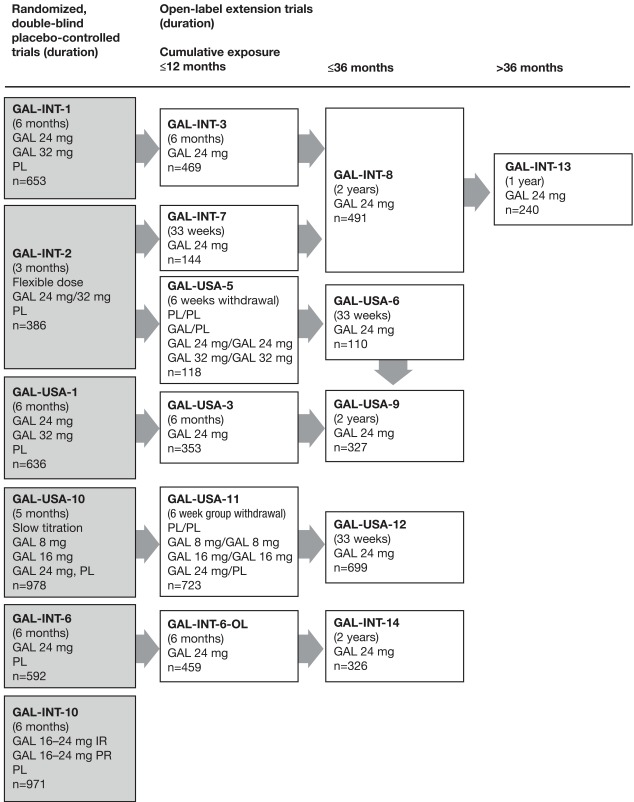
Overview of the 6 randomized, double-blind, placebo-controlled trials of galantamine (GAL) in patients with Alzheimer’s disease (shaded boxes) and the subsequent open-label extensions included in the analyses. IR, immediate-release formulation (twice daily); n, number of patients starting the trial; PL, placebo; PR, prolonged-release formulation (once daily). Note that 18 patients from GAL-USA-10 entered GAL-USA-12 without first completing GAL-USA-11. Also, 285 patients in GAL-INT-6 had Alzheimer’s disease with concomitant cerebrovascular disease and were included in the analyses; the remaining 307 patients in this trial had probable vascular dementia and were excluded from the analyses.

Analyses included data from 6 double-blind, placebo-controlled RCTs in patients with mild to moderate AD (AD+CVD in GAL-INT-6), in which currently recommended maintenance doses of galantamine (16-24 mg/day) were administered [3-8] (Fig. **1**). All studies were carried out according to the Declaration of Helsinki and subsequent revisions, and were approved by institutional review boards or ethics committees.

All 6 studies enrolled patients with a history of cognitive decline; all except 1 study (GAL-INT-6) required that this be gradual in onset and progressive for at least 6 months. In all studies (except GAL-INT-6), patients met National Institute of Neurological and Communicative Disorders and Strokeand the Alzheimer’s Disease and Related Disorders Association (NINCDS-ADRDA) [13] criteria for probable AD. Patients in study GAL-INT-6 met either NINCDS-ADRDA criteria for possible AD or National Institute of Neurological Disorders and Stroke and Association Internationale pour la Recherche et l’Enseignement en Neurosciences (NINDS-AIREN) International Workshop [14] criteria for probable vascular dementia (VAD). For all studies, eligible patients were required to have baseline Mini-Mental State Examination (MMSE) [15] scores of 10–25. Baseline scores on the 11-item Cognitive subscale of the Alzheimer’s Disease Assessment Scale (ADAS-Cog) [16] had to be ≥12 in all except two studies where it had to be ≥18. The reported mean ages for all study groups ranged from 72-78 years.

In one study (GAL-INT-10), both immediate- and prolonged-release galantamine arms were included in the analysis. In a flexible-dose trial (GAL-INT-2), which used a galantamine arm of 24 or 32 mg daily, all galantamine-treated patients were included.

Only the subgroup of patients (~50%) with AD+CVD were included from the GAL-INT-6 study, providing data for an AD population with a comorbidity commonly observed in clinical practice. Patients with VAD were excluded.

Data from patients who received galantamine 8 mg/day (GAL-USA-10 [n=140]) or 32 mg/day (GAL-INT-1 [n=218] or GAL-USA-1 [n=211]) maintenance doses, and who could progress to the open-label phase, were excluded from the responder analyses described in this paper.

Data from 12 long-term, open-label extensions of the RCTs, some of which have been published [4, 9-11] were included (Fig. **1**) to determine whether the initial response to galantamine was predictive for long-term response. All long-term extension trials used galantamine 24 mg/day as the maintenance dosage, with systematic follow-up using standardized scheduled assessments of tolerability and efficacy.

### Responder Definitions

ADAS-Cog 11 was used to measure cognitive response. A negative change on this scale indicates improvement in cognition, whereas a positive change indicates deterioration. For our analyses, the following (non-mutually exclusive) definitions were used:

#### Responder 1 — Improved

Stable or improved cognition (ADAS-Cog 11 score change from baseline ≤0) AND improved global assessment (Clinician’s Interview-Based Impression of Change with Caregiver Input [CIBIC-plus] or Clinician’s Global Impression of Change [CGIC]); OR function (AD Cooperative Study of Activities of Daily Living Inventory [ADCS-ADL] or Disability Assessment for Dementia [DAD]); OR behavior (Neuropsychiatric Inventory [NPI]).

#### Responder 2 — Stable

Stable or improved cognition (ADAS-Cog 11 score change from baseline ≤0) AND stable or improved global assessment (CIBIC-plus or CGIC), OR function (ADCS-ADL or DAD), OR behavior (NPI).

#### Responder 3 — Non-Rapid Decline

Improvement, stability, or minimal deterioration in cognition (ADAS-Cog 11 score change from baseline ≤4 points). This group of patients therefore excludes only those whose cognition declines rapidly.

Each trial used 1 global (CIBIC-plus or CGIC) and 1 functional (ADCS-ADL or DAD) assessment. In some trials where NPI data were not collected, analyses were limited to cognition, function and global scores in terms of estimating improvement and/or stabilization.

### Analyses

To assess the short-term efficacy of galantamine, data from the 4 RCTs (GAL-INT-1, GAL-INT-10, GAL-USA-1 and GAL-USA-10) in patients with probable AD considered to be of adequate duration to ascertain effectiveness (assessments 2-5 months after attainment of maintenance dose) were pooled using simple pooled analysis with no covariates. Data are also presented separately for these trials, as well as for the subgroup of patients with possible AD+CVD from GAL-INT-6.

Three sets of analyses were performed, enabling evaluation not only of patients who completed the trials, but also including individuals who withdrew from the studies. The observed-cases (OC) analysis was based on changes in scores (from baseline) for patients who completed the trials and for whom the relevant instruments were sufficiently complete to allow calculation of change scores. The last observation carried forward (LOCF) analysis included patients who completed at least 1 post-baseline assessment (i.e., the intent-to-treat population), and, for those who withdrew, the measurement from the last (post-baseline) assessment available was carried forward to allow calculation of score changes. The observed cases plus (OC+) analysis was based on observed cases plus an LOCF determination for a small number of patients who discontinued due to lack of efficacy. Particular attention was given to identifying patients who discontinued due to lack of efficacy. In the first instance, category responses recorded on the study termination form for “inefficacy” and “insufficient response” (n=18) were utilized. To ensure completeness, an additional search was conducted of individual verbatim comments on the trial termination form for other general categories such as “subject withdrew consent” and “other”, that led to the identification of a further 14 cases (32 in total, <1%) in which inefficacy or similar wording was mentioned. In common with LOCF analyses, such patients were included if they had at least 1 post-baseline assessment (n=20).

To evaluate long-term efficacy, ADAS-Cog 11 changes for patients with probable AD who originally participated in the 5-6 month trials and the subsequent open-label extensions (Fig. **1**) were assessed for up to 48 months post-treatment initiation in both short-term responders and non-responders. Analyses were repeated including AD+CVD patients. These data were compared with the predicted change in cognition for untreated patients using the Stern equation estimates of ADAS-Cog 11 decline [17]. The Stern prediction of decline was used as a benchmark for the expected long-term untreated deterioration in cognition, as measured by the ADAS-Cog 11 score in the absence of treatment. The Stern model takes into account the baseline ADAS-Cog 11 score and uses the time from that score to predict the longitudinal rate of decline [17]. A quadratic relationship has been demonstrated between the severity of AD and the rate of cognitive decline, with good agreement between observed and predicted changes [12]. To account for potential dropout bias, an additional corrected Stern estimate based on the baseline characteristics of patients available at each assessment point was also used.

## RESULTS

A total of 3,523 patients participated in the 5 RCTs of 5-6 months’ duration; this included 285 patients with AD+CVD in GAL-INT-6, but excluded 307 patients in the same trial with VAD. Reasons for discontinuation from these trials included adverse events (11%), withdrawal of consent (3%), noncompliance (2%), loss to follow-up (<1%), insufficient response/inefficacy (<1%), ineligible to continue (<1%), death (<1%), and other (4%).

Following the RCTs, a total of 2,266 patients subsequently entered the open-label extension trials. Reasons for discontinuation during the open-label extensions included adverse events (21%), withdrawal of consent (7%), death (4%), insufficient response/inefficacy (3%), noncompliance (2%), loss to follow-up (1%), did not enter Year 2 (1%), ineligible to continue (<1%), and other (8%). After completing one open-label trial, 5% of patients did not enter a further open-label study for which they were subsequently eligible. The reasons for eligible patients not proceeding from one open-label study to the next were not available.

### Short-Term Treatment Response

Analysis of the total pooled probable AD (i.e., excluding AD+CVD) 5- to 6-month trial population (OC analyses) showed that galantamine-treated patients improved by 1.5 points (vs. baseline) on the ADAS-Cog 11 scale, whereas the placebo group deteriorated by 1.8 points (overall galantamine treatment benefit, 3.3 points; p<0.001) (Table **1**). (In patients with possible AD+CVD, overall treatment benefit was 2.8 points on the ADAS-Cog 11 scale [p<0.001].)

### Proportion of Responders: “Responder 1 — Improved” Definition

Among patients with probable AD, 45.8% of galantamine-treated patients met the “Responder 1 — improved” criteria compared with 27.2% of placebo recipients. (In patients with possible AD+CVD, 47.4% and 35.6% of the galantamine and placebo groups, respectively, met the “Responder 1 — improved” criteria).

The magnitude of improvement on the ADAS-Cog 11 scale was greater for patients meeting the “Responder 1 — improved” criteria (4.9 points) compared with the overall galantamine group (1.5 points) (Table **1**).

The other analyses (OC+ and LOCF) showed broadly consistent findings (Table **1**). Because relatively few patients discontinued due to lack of efficacy, results for the OC and OC+ analyses were almost identical in terms of the proportions of patients meeting the “Responder 1 — improved” criteria as well as in the magnitude of changes in ADAS-Cog 11 scores from baseline.

**Fig. (2):**
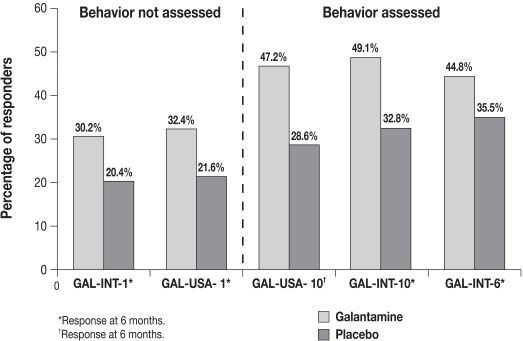
Responder rates (“Responder 1 — improved” definition: stable/improved cognition, and improved global assessment or function or behavior) for galantamine- and placebo-treated patients in individual trials of 5-6 months’ duration (LOCF analysis). LOCF analysis is defined as the intent-to-treat analysis with missing data for patients who discontinued, computed as the last observation carried forward.

“Responder 1” rates were higher in trials in which behavior was measured (Fig. **2**). In the LOCF analysis, the proportions of galantamine-treated patients fulfilling the criteria for “Responder 1 — improved” ranged from 44.8% to 49.1% compared with 30.2% to 32.4% in trials without a behavioral measure. Similar differences in “Responder 1” rates for galantamine-treated patients in trials with or without a behavioral measure were noted for the OC and OC+ analyses (data not shown).

### Proportion of Responders: “Responder 2 — Stable” Definition

The proportion of probable AD patients meeting the “Responder 2 — stable” criteria was 59.5% with galantamine versus 37.1% with placebo (OC analysis) (Table **2**). (In patients with possible AD+CVD, “Responder 2” rates were consistent, with 52.7% and 39.1% for the galantamine and placebo groups, respectively.) The proportions of patients meeting the “Responder 2 — stable” criteria in the OC+ and LOCF analyses were consistent with these findings (data not shown). Overall, when this “stable” definition of response was applied, the magnitude of treatment change in the ADAS-Cog 11 score at endpoint in both galantamine and placebo groups was similar to that when the “Responder 1 — improved” criteria were used (Table **1**) e.g., -4.9 points on the ADAS-Cog 11 scale in the galantamine “Responder 1” group versus -4.7 points in the galantamine “Responder 2” group (Tables **1** and **2**).

### Proportion of Responders: “Responder 3 —Non-Rapid Decline” Definition 

The proportion of patients with probable AD who met the “Responder 3 — non-rapid decline” criteria was 87.6% with galantamine versus 69.7% with placebo (OC analysis) (Table **2**). (In patients with possible AD+CVD, “Responder 3” rates were 82.9% and 72.4% for galantamine and placebo, respectively.) The proportions of patients meeting “Responder 3 — non-rapid decline” criteria in the OC+ and LOCF analyses were consistent with these findings (data not shown). Overall, when this “non-rapid decline” definition of response was used, the magnitude of change in the ADAS-Cog 11 score versus baseline at endpoint was -2.9 points, somewhat lower compared to “Responder 1” (-4.9 points) and “Responder 2” (-4.7 points), but still greater than for the treatment group as a whole (-1.5 points) (Tables **1** and **2**). 

### Long-Term Effects in Responders and Non-Responders

**Fig. (3):**
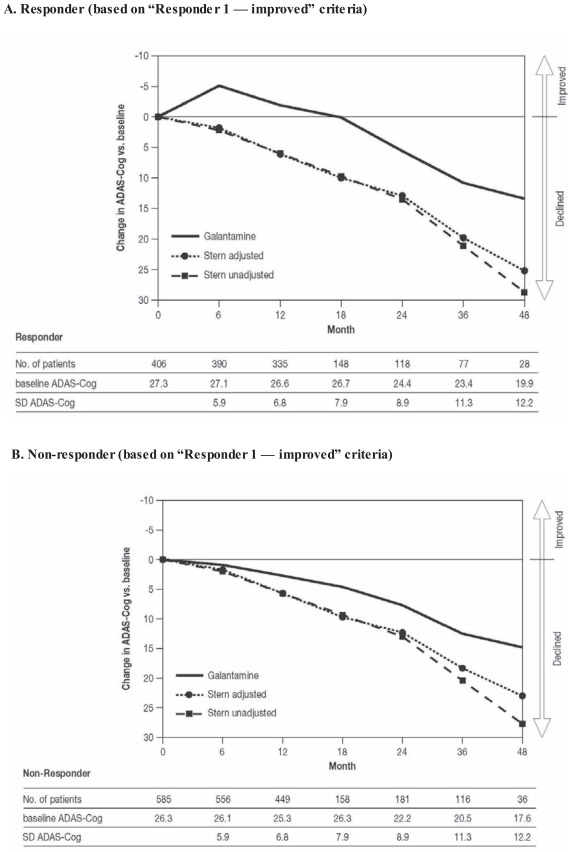
Long-term changes in scores on the 11-item Alzheimer’s Disease Assessment Scale–Cognitive subscale (ADAS-Cog 11) for responders to galantamine (**A**), defined as “improved” (“Responder 1” criteria: stable/improved cognition, and improved global assessment or function or behavior) at 5-6 months, compared with the Stern prediction [17] of untreated decline (unadjusted and adjusted for the baseline characteristics of patients available at each assessment point). Non-responders are also shown (**B**).

Galantamine-treated patients with probable AD who met the “Responder 1— improved” criteria displayed a mean improvement in ADAS-Cog 11 scores versus baseline until 18 months after initiation of treatment, with an attenuated rate of deterioration thereafter (Fig. **3A**). In galantamine-treated patients who met the “Responder 1” non-response criteria, the deterioration in mean ADAS-Cog 11 scores was somewhat less than for the Stern prediction of untreated decline (adjusted for the dropout of more advanced patients over time) (Fig. **3B**).

**Fig. (4):**
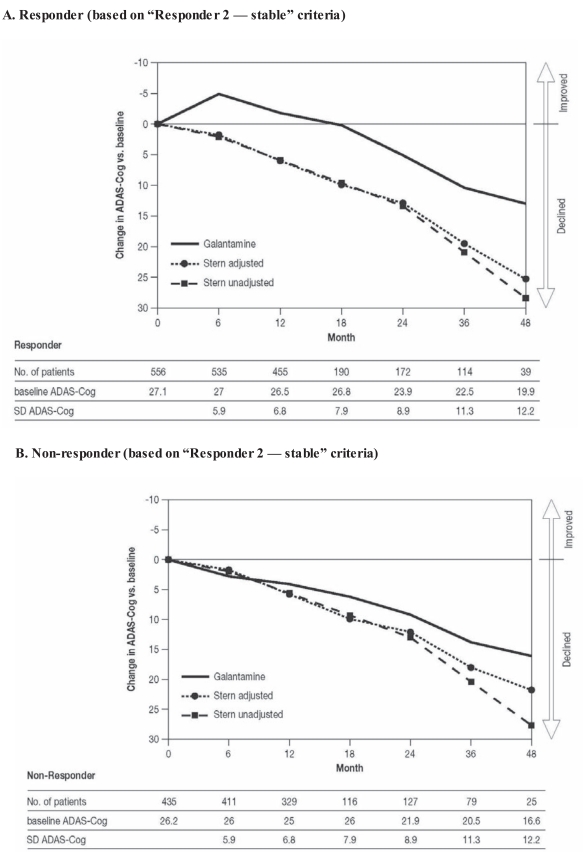
Long-term changes in scores on the 11-item Alzheimer’s Disease Assessment Scale–Cognitive subscale (ADAS-Cog 11) for responders to galantamine (**A**), defined as “stable” (“Responder 2” criteria: stable/improved cognition, and stable/improved global assessment or function or behavior) at 5-6 months, compared with the Stern prediction [17] of untreated decline (unadjusted and adjusted for the baseline characteristics of patients available at each assessment point). Non-responders are also shown (**B**).

Similarly, when the “Responder 2 — stable” group was considered, there was also a mean improvement in ADAS-Cog 11 scores versus baseline for galantamine-treated patients until 18 months after initiation of treatment, with a similarly attenuated rate of deterioration thereafter (Fig. **4A**). The observed deterioration in mean ADAS-Cog 11 scores among galantamine-treated patients who met the “Responder 2” non-response criteria was closer to the Stern-predicted untreated deterioration (Fig. **4B**), although some individual patients in this group outperformed their Stern-predicted untreated decline.

**Fig. (5):**
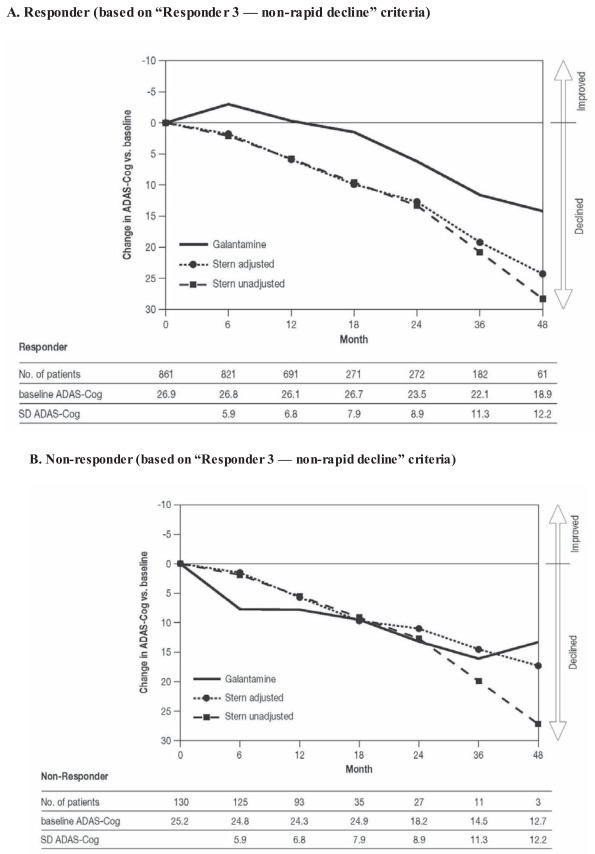
Long-term changes in scores on the 11-item Alzheimer’s Disease Assessment Scale–Cognitive subscale (ADAS-Cog 11) for responders to galantamine (**A**), defined as “non-rapid decline” (“Responder 3” criteria: improvement, stability, or minimal deterioration in cognition) at 5-6 months, compared with the Stern prediction [17] of untreated decline (unadjusted and adjusted for the baseline characteristics of patients available at each assessment point). Non-responders are also shown (**B**).

For galantamine-treated patients with probable AD who met the “Responder 3 — non-rapid decline” definition, approximately 12 months elapsed before the mean recorded ADAS-Cog 11 score returned to the baseline level. Beyond 12 months, the observed deterioration was less than the Stern-predicted untreated decline (Fig. **5A**). For galantamine-treated patients who met the “Responder 3” non-response criteria, the observed mean deterioration in ADAS-Cog 11 scores was very similar to the Stern-predicted untreated decline (Fig. **5B**), and relatively few individual patients who met the non-response criteria outperformed their Stern-predicted untreated decline.

Consistent results were obtained across all three responder definitions when analyses were conducted that also included patients with AD+CVD (data not shown).

## DISCUSSION

The results of the present pooled analyses suggest that patients who demonstrate improvement, stability on assessments of cognition and other domains such as function/behavior, or non-rapid cognitive decline 5-6 months after the initiation of galantamine therapy are more likely to experience continued benefit from long-term therapy. Patients who exhibit rapid cognitive decline at 5-6 months (such as >4 points on the ADAS Cog-11 scale or >2 points on the MMSE) are likely to be non-responders to long-term galantamine therapy. It is thus possible that currently used definitions of response that require improvement may result in the exclusion of a substantial proportion of patients who could potentially benefit from longer-term treatment. The current analyses suggest that patients with “stable” and to a lesser extent “non-rapid decline” definitions of response may also benefit from galantamine therapy in the long term. However, when considering the data presented in this analysis, it is important to take into account both the strengths and weaknesses of our study.

The retention of elderly and frail patients (and their caregivers — who may also be elderly and infirm) in clinical trials is a major challenge. Nevertheless, retention rates in the present long-term trials were reasonably satisfactory and so data were available for >1,100 patients continuously treated with galantamine for 12 months, >500 treated for 24 months, and >300 treated for 36 months. (As mentioned earlier, patients who had previously received galantamine 8 mg/day [in study GAL-USA-1032] or 32 mg/day [in studies GAL-INT-1 or GAL-USA-1] were excluded from the responder analyses).

The discontinuation rate in the first 6 months due to lack of efficacy was very low (<1%), even when trial databases were “hand-searched” for such patients. Therefore, inclusion of such patients likely had little impact on the analysis. In line with the double-blind trials, the data from the open-label extension studies — the original objectives of which were to examine the long-term safety of galantamine rather than long-term efficacy — showed that most patients who discontinued did so as a consequence of adverse events; lack of efficacy was given as the reason for discontinuation from the open-label extension studies in only 3% of instances. Nevertheless, selection effects with trial withdrawals cannot be precluded. The substantial drop in patient numbers observed in the present analysis after 12 months should also be acknowledged. To control for the possibility of selective dropouts, as previously mentioned, a corrected Stern estimate, based on the baseline characteristics of patients available at each assessment point, was used when evaluating the data.

The Stern equation was used in the absence of placebo data and we accept that the analysis of these trials was limited by the lack of a long-term placebo group. Recruitment and long-term retention of a meaningful placebo group was precluded for both ethical and practical reasons due to the availability of established licensed medications for AD. (Indeed, the availability of licensed medications for AD has affected patient recruitment/retention in other studies [18] and may result in newly developed drugs with potential disease-modifying effects [19] being administered as adjuvant therapies.) Under these circumstances, long-term placebo groups might not have provided representative comparators. Whilst the Stern equation provides a useful benchmark based on epidemiological modeling of long-term deterioration in untreated patients, the limitations of such methodology should be noted — these limitations include deriving estimates from data involving relatively small numbers of patients. Comparison of the Stern equation with 1-year placebo data from two clinical trials of patients with mild to moderate AD showed a reasonably good level of agreement in the overall population [20]. However, the predicted decline in patients with moderate AD was less than observed in the trial data, suggesting that the Stern estimates may be conservative [20], and predictions in subgroups of patients should be interpreted cautiously [21].

We consider that the “Responder 2 — stable” criteria may be regarded as the best performing of the three “Responder” definitions we have described. External review of these data [21], which were originally submitted to the National Institute for Health and Clinical Excellence (NICE) in England and Wales, suggested that the “Responder 2 — stable” criteria were not significantly affected by potential regression to the mean and that the “Responder 2 — stable” criteria did not lead to significant diminution in treatment effect compared to “Responder 1 — improved” criteria. With the “Responder 3 — non-rapid decline” criteria, a higher proportion of placebo responders was apparent: the proportions of placebo responders after 5-6 months according to “Responder 1 — improved”, “Responder 2 — stable” and “Responder 3 — non-rapid decline” criteria were 27.2, 37.1 and 69.7%, respectively (OC analyses). Given the higher proportion of placebo responders with the “Responder 3” criteria, a greater degree of regression to the mean might have been expected in this instance. However, with the “Responder 3” criteria, the mean baseline ADAS-Cog 11 score was higher in placebo non-responders than in placebo responders (29.0 vs 24.4 points), and this should have resulted in a greater effect in placebo non-responders if regression to the mean was exerting a substantial effect. Furthermore, if the effects of regression to the mean had influenced the results, decline in non-responding patients may have been underestimated, whilst for responders changes could have been evaluated conservatively. This would strengthen our argument relating to potential benefits of galantamine therapy. (For all definitions of response, mean cognition scores at baseline were comparable between responders and non-responders in the galantamine-treated patients, and we feel that regression to the mean was likely to have been very limited in these patient groups).

Despite the limitations of our study, the presented data are the best currently available and provide useful insights into the long-term outcome of galantamine treatment. With respect to clinicians, our results suggest that consideration should be given to continuing galantamine in patients who exhibit stable cognition 2-5 months after reaching maintenance doses of galantamine. Furthermore, these data support consideration for discontinuation of galantamine treatment in patients who exhibit rapid decline 2-5 months after reaching maintenance doses.

In conclusion, when patients commence galantamine therapy, the potential benefits are unknown. However, identifying and continuing to treat patients who demonstrate improvement, stability, or limited cognitive decline (ADAS-Cog 11 score change from baseline ≤4 points) 2-5 months after reaching maintenance doses of galantamine may allow more patients to experience the benefits of long-term galantamine therapy.

## Figures and Tables

**Table 1:** Effects of Galantamine on Cognition (ADAS-Cog 11 Scale) in Pooled AD Trials Using the “Responder 1 — Improved” Criteria (Stable or Improved Cognition, and Improved Global Assessment or Function or Behavior)^a^ for Responders at Endpoint (5-6 Months)

	No. of Patients (at Baseline)	Mean Cognition Score (SD) at Baseline [95% CI]	No. (%) of Patients at Endpoint	Mean Cognition Score (SD) at Endpoint [95% CI]	Mean Change in Cognition Score (SD) from Baseline to Endpoint^b^ [95% CI]
**OC Analysis**					
*Galantamine (16-24 mg)*					
Total	1,173	26.6 (9.8) [26.1, 27.2]	1,173	25.1 (10.9) [24.5, 25.7]	-1.5 (5.9) [-1.8, -1.2]
Responders	NA	27.2 (9.5) [26.4, 28.0]	537 (45.8)	22.3 (9.5) [21.5, 23.1]	-4.9 (4.1) [-5.3, -4.6]
Non-responders	NA	26.1 (10.0) [25.3, 26.9]	636 (54.2)	27.5 (11.4) [26.6, 28.4]	1.4 (5.6) [0.9, 1.8]
*Placebo*					
Total	801	25.8 (9.7) [25.1, 26.5]	801	27.6 (12.4) [26.8, 28.5]	1.8 (6.1) [1.4, 2.3]
Responders	NA	23.5 (8.5) [22.4, 24.7]	218 (27.2)	20.0 (8.5) [18.8, 21.1]	-3.6 (3.0) [-4.0, -3.2]
Non-responders	NA	26.6 (10.0) [25.8, 27.4]	583 (72.8)	30.5 (12.4) [29.5, 31.5]	3.9 (5.7) [3.4, 4.3]
**OC+ Analysis**					
*Galantamine (16-24 mg)*					
Total	1,183	26.6 (9.8) [26.1, 27.2]	1,183	25.1 (10.8) [24.5, 25.7]	-1.5 (5.9) [-1.8, -1.2]
Responders	NA	27.2 (9.5) [26.4, 28.0]	541 (45.7)	22.3 (9.4) [21.5, 23.1]	-4.9 (4.1) [-5.3, -4.6]
Non-responders	NA	26.1 (10.0) [25.3, 26.9]	642 (54.3)	27.5 (11.4) [26.6, 28.4]	1.4 (5.6) [1.0, 1.8]
*Placebo*					
Total	811	25.9 (9.8) [25.2, 26.5]	811	27.7 (12.4) [26.9, 28.6]	1.8 (6.1) [1.4, 2.3]
Responders	NA	23.6 (8.5) [22.5, 24.7]	220 (27.1)	20.0 (8.5) [18.9, 21.2]	-3.6 (3.0) [-4.0, -3.2]
Non-responders	NA	26.7 (10.1) [25.9, 27.5]	591 (72.9)	30.6 (12.4) [29.6, 31.6]	3.9 (5.7) [3.4, 4.3]
**LOCF Analysis**					
*Galantamine (16-24 mg)*					
Total	1,466	27.0 (9.9) [26.5, 27.5]	1,466	25.6 (10.9) [25.1, 26.2]	-1.4 (5.7) [-1.6, -1.1]
Responders	NA	27.3 (9.6) [26.6, 28.1]	644 (43.9)	22.6 (9.5) [21.8, 23.3]	-4.7 (4.0) [-5.0, -4.4]
Non-responders	NA	26.7 (10.2) [26.0, 27.4]	822 (56.1)	28.0 (11.4) [27.2, 28.8]	1.3 (5.4) [0.9, 1.7]
*Placebo*					
Total	951	26.4 (10.1) [25.8, 27.1]	951	28.1 (12.5) [27.3, 28.9]	1.7 (6.0) [1.3, 2.1]
Responders	NA	24.3 (8.8) [23.2, 25.4]	254 (26.7)	20.6 (8.8) [19.5, 21.7]	-3.7 (3.1) [-4.1, -3.3]
Non-responders	NA	27.2 (10.5) [26.4, 28.0]	697 (73.3)	30.9 (12.5) [30.0, 31.8]	3.7 (5.6) [3.3, 4.1]

a At 5 to 6 months: Change in ADAS-Cog 11 scores was ≤0 AND there was improved global (CIBIC-plus or CGIC) OR functional (ADCS-ADL or DAD) OR behavioral assessment (NPI). Each trial used 1 global assessment (either CIBIC-plus or CGIC) and 1 functional assessment (either ADCS-ADL or DAD). In a number of trials, NPI data were not collected.

b A negative change in the score indicates improvement; a positive change indicates deterioration. AD, Alzheimer’s disease; ADAS-Cog 11, 11-item Alzheimer’s Disease Assessment Scale–Cognitive subscale; ADCS-ADL, AD Cooperative Study of Activities of Daily Living Inventory; CGIC, Clinician’s Global Impression of Change; CI, confidence interval; CIBIC-plus, Clinician’s Interview-Based Impression of Change with Caregiver Input; DAD, Disability Assessment for Dementia; LOCF, intent-to-treat analysis with missing data for patients who discontinued, computed as the last observation carried forward; NPI, Neuropsychiatric Inventory; OC, observed cases; OC+, OC analysis with LOCF for patients who discontinued due to lack of efficacy; SD, standard deviation; NA, not applicable.

**Table 2:** Effects of Galantamine on Cognition (ADAS-Cog 11 Scale) in AD Pooled Trials Using “Responder 2 — Stable” (Stable or Improved Cognition, and Stable or Improved Global Assessment or Function or Behavior)^a^ or “Responder 3 — Non-rapid Decline” (Improvement, Stability, or Minimal Deterioration in Cognition)^b^ Criteria for Responders at Endpoint (5-6 Months)

	No. of Patients (at Baseline)	Mean Cognition Score (SD) at Baseline [95% CI]	No. (%) of Patients at Endpoint	Mean Cognition Score (SD) at Endpoint [95% CI]	Mean Change in Cognition Score (SD) from Baseline to Endpoint^c^ [95% CI]
**“Stable” Response Criteria**^a^** (OC analysis)**					
*Galantamine (16-24 mg)*					
Total	1,173	26.6 (9.8) [26.1, 27.2]	1,173	25.1 (10.9) [24.5, 25.7]	-1.5 (5.9) [-1.8, -1.2]
Responders	NA	26.8 (9.5) [26.1, 27.5]	698 (59.5)	22.1 (9.3) [21.4, 22.8]	-4.7 (4.0) [-5.0, -4.4]
Non-responders	NA	26.4 (10.2) [25.5, 27.3]	475 (40.5)	29.6 (11.4) [28.6, 30.6]	3.2 (5.0) [2.7, 3.6]
*Placebo*					
Total	801	25.8 (9.7) [25.1, 26.5]	801	27.6 (12.4) [26.8, 28.5]	1.8 (6.1) [1.4, 2.3]
Responders	NA	23.3 (8.5) [22.3, 24.3]	297 (37.1)	19.8 (8.4) [18.9, 20.8]	-3.4 (3.0) [-3.8, -3.1]
Non-responders	NA	27.2 (10.1) [26.4, 28.1]	504 (62.9)	32.2 (12.0) [31.1, 33.2]	5.0 (5.3) [4.5, 5.4]
**“Non-Rapid Decline” Response Criteria**^b^** (OC Analysis)**					
*Galantamine (16-24 mg)*					
Total	1,173	26.6 (9.8) [26.1, 27.2]	1,173	25.1 (10.9) [24.5, 25.7]	-1.5 (5.9) [-1.8, -1.2]
Responders	NA	26.5 (9.8) [25.9, 29.2]	1,028 (87.6)	23.6 (9.9) [23.0, 24.2]	-2.9 (4.6) [-3.2, -2.6]
Non-responders	NA	27.6 (9.9) [25.9, 29.2]	145 (12.4)	36.1 (11.2) [34.2, 37.9]	8.5 (4.1) [7.8, 9.2]
*Placebo*					
Total	801	25.8 (9.7) [25.1, 26.5]	801	27.6 (12.4) [26.8, 28.5]	1.8 (6.1) [1.4, 2.3]
Responders	NA	24.4 (9.1) [23.6, 25.1]	558 (69.7)	23.1 (9.8) [22.3, 23.9]	-1.2 (3.8) [-1.6, -0.9]
Non-responders	NA	29.0 (10.4) [27.7, 30.3]	243 (30.3)	37.9 (11.5) [36.5, 39.4]	8.9 (4.3) [8.4, 9.5]

a At 5 to 6 months: Change in ADAS-Cog 11 scores was ≤0 AND there was stable/improved global (CIBIC-plus or CGIC) OR functional (ADCS-ADL or DAD) OR behavioral assessment (NPI). Each trial used 1 global assessment (either CIBIC-plus or CGIC) and 1 functional assessment (either ADCS-ADL or DAD). In a number of trials, NPI data were not collected.

b At 5 to 6 months: Patient either improved, stable, or showed a deterioration of 4 points or fewer on the ADAS-Cog 11 scale.

c A negative change in the score indicates improvement; a positive change indicates deterioration. AD, Alzheimer’s disease; ADAS-Cog 11, 11-item Alzheimer’s Disease Assessment Scale–Cognitive subscale; ADCS-ADL, AD Cooperative Study of Activities of Daily Living Inventory; CGIC, Clinician’s Global Impression of Change; CI, confidence interval; CIBIC-plus, Clinician’s Interview-Based Impression of Change with Caregiver Input; DAD, Disability Assessment for Dementia; NPI, Neuropsychiatric Inventory; OC, observed cases; SD, standard deviation; NA, not applicable.
